# Mammary gland-derived nestin-positive cell populations can be isolated from human male and female donors

**DOI:** 10.1186/scrt229

**Published:** 2013-07-08

**Authors:** Anja Richter, Nicole Nissen, Peter Mailänder, Felix Stang, Frank Siemers, Charli Kruse, Sandra Danner

**Affiliations:** 1 Department of Neurology, Neurobiochemistry Group, University of Luebeck, Ratzeburger Allee 160, Luebeck 23538, Germany; 2 Fraunhofer Research Institution for Marine Biotechnology, Paul-Ehrlich-Straße 1-3, Luebeck, Germany; 3 Clinic for Plastic, Hand and Reconstructive Surgery, Luebeck University Clinics, Ratzeburger Allee 160, Luebeck 23562, Germany

**Keywords:** Mammary gland, Adult stem cells, Nestin, Isolation, Multilineage potential

## Abstract

**Introduction:**

Nestin-expressing cells isolated from different human tissues reveal self-renewal capacity and a multilineage differentiation potential. In particular, adult stem/progenitor cell populations from exocrine glands such as the pancreas, salivary gland and sweat gland are characterized by prominent nestin expression. Interestingly, human mammary gland histological examinations also demonstrated the existence of nestin-positive cells in the ductal compartments. Within the scope of our previous work we wonder whether an isolation of nestin-positive cell populations from human mammary gland biopsies is possible and what characteristics they have *in vitro*. Cell populations from both sexes were propagated and subjected to a comparison with other gland-derived cell populations.

**Methods:**

Human mammary tissue biopsies were mechanically and enzymatically treated, and the isolated acini structures were observed with time-lapse microscopy to track adherently outgrowing cells. The proliferation potential of the cell population was assessed by performing growth curves. On the gene and protein levels we investigated the expression of stem cell markers as well as markers indicating multilineage differentiation.

**Results:**

We succeeded in establishing proliferating cell populations from breast tissue biopsies of both sexes. Our results display several similarities to the glandular stem cell populations from other exocrine glands. Beside their proliferation capacity during *in vitro* culture, the obtained cell populations are characterized by their prominent nestin expression. The cells share surface proteins commonly expressed on adult stem cells. We demonstrated the expression of stem cell-related genes like Oct4, Sox2, KLF4 and Nanog, and confirmed multipotent differentiation capacity by detecting transcripts expressed in endodermal, mesodermal and ectodermal cell types.

**Conclusion:**

With this study we present an efficient procedure for isolation and propagation of nestin-positive stem cells obtained from male and female breast tissue, which is frequently available. The established multipotent cell populations could be easily expanded *in vitro* and thus hold promise for cell-based therapies and personalized medicine.

## Introduction

Potential stem/progenitor cell populations in the mammary gland were the subject of a variety of research projects with the aim to understand gland development, maturation, lactation as well as tumor formation. Owing to the constant evolution of new techniques for analysis, isolation, cultivation and transplantation, the knowledge about mammary stem/progenitor cells increased continuously over the years. To understand the behavior of adult stem cells *in vivo* and *in vitro*, it is necessary to address briefly the normal human gland development, with a focus on the key mechanisms that are thought to be important in human and rodents.

The embryonic mammary placodes contain ectodermal epithelium (keratin (K)14-positive) involuted into mesodermal stroma. The so-called mammary bud is filled with multipotent progenitors (K5-positive and K14-positive) and forms until birth a small, less branched, resting gland precursor below the nipple. The cellular topography of the gland is established at that point by a basal K14-positive and K5-positive myoepithelial layer and a luminal layer synthesizing cytokeratins 8 and 19. A subpopulation of K14-positive and K8-positive luminal cells is interwoven into the luminal layer while fewer cells still synthesize K5. In resting glands after complete juvenile development, only K8/19-positive luminal cells and K14/15-positive myoepithelial cells exist [1-4].

The mammary gland maturation during puberty includes the formation of so-called terminal end buds at the tips of the branches. The main signaling for this event is mediated by hormones (for example, endothelial growth factor) and estrogen [5]. The terminal end buds consist of body cells enveloped by cap cells. The latter are thought to be a tissue-specific type of stem/progenitor cell and the source of luminal epithelial cells and myoepithelial cells [5]. Both cell types form in a typical epithelial fashion the hollow branches of the mammary gland that extend up to the nipple conducting the milk. During pregnancy and lactation the mammary gland sprouts extensively into the breast connective tissue. For milk secretion, lobular cells at the ends of the branches form secretory acinar structures and transform to alveolar cells producing milk proteins [5,6]. These luminal cell-derived milk producing cells synthesize K8 and K19 [1].

The enormous tissue expansion during pregnancy somehow needs proliferative active cell populations, which ensure endogenous tissue formation in adulthood. There is still controversy about the existence of unipotent, bipotent or multipotent stem cells in mammary gland tissue [1-3].

The existence and isolation of stem cells in mammary glands was described by several groups starting decades ago. In 1959 Deome and colleagues described mice transplantation studies of mammary tissue into body sites freed of this tissue, resulting in a successful reestablishment of mammary outgrows [2]. In 1988 the identification of label-retaining cells in mammary explant cultures from mouse using thymidine labeling was demonstrated by Smith and Medina [7]. The isolation of tissue-resident stem cells is nowadays commonly performed by enzymatic digestion and/or by flow cytometric isolation of subpopulations using surface markers. For instance, Shackelton and colleagues demonstrated the isolation of Lin^–^CD29^hi^CD24^+^ cells from mouse mammary tissue and their power to reconstitute a complete mammary gland in cleared mammary fad pads, even when they transplanted just one cell [3]. Eirew and colleagues revealed a CD49f^+^ and EpCAM^neg-low^ phenotype for human mammary repopulating units by investigating a subset of cells that give rise to newly formed mammary glands in serial transplantation experiments similar to those performed in the 1950s [8].

A relatively broad spectrum of potent surface markers and their usage for isolation of stem/progenitor cells from mammary glands via cell sorting has been investigated and discussed in the literature [1,3,9-13]. Prominent candidates such as Sca-1, EpCAM, CD14, CD24, CD29, CD49f and CD61 for mouse and human mammary stem or progenitor populations were reviewed by Stingl and colleagues in 2006 [10]. In congruence with the earlier mentioned native developmental function, these results of transplantation studies with certain mammary stem cell subpopulations indicated their multipotency by giving rise to mammary committed progenitors with progeny of different fates [8,9,11,13-15]. To clarify this cellular plasticity, various *in vitro* assays of mammary-derived cell populations had been performed. First insights into the characteristics and behavior of isolated mammary stem cells *in vitro* were gained from adherent two-dimensional cultures or from suspension cultures; for example, as mammospheres. The focus of gene and protein expression analysis for those studies was chosen for surface markers and/or cytokeratin synthesis, to classify the state of differentiation in comparison with histological data [11,13-21]. The results demonstrated that the mammary stem cells preserve their capability for differentiation into mammary cells and retain the sensitivity against tissue-specific hormones [16,22]. On the contrary, their continuous proliferation and the expression of stem cell and proliferation-related markers such as Ki67, musashi-1, Sca-1 or p21 affirmed that they keep a stem/progenitor state [11,13-18]. Interestingly, even the isolation and cultivation of stem cells from human breast milk has been documented [19-21]. Hassiotou and colleagues recently demonstrated the isolation of cell populations from human breast milk, which expressed stem cell-related transcription factors such as Oct4, Sox2 and Nanog and proved their multilineage differentiation potential [19].

A less popular marker for the characterization of mammary stem cells is nestin, which has been expressed in cells of the mammary gland [23,24]. The expression of the intermediate filament nestin has already been shown to be associated with multipotency and stemness of several cell populations [25]. We and others demonstrated that different glandular tissues (for example, pancreas, salivary glands or sweat glands) yield nestin-positive stem cell populations with multipotent, long-term proliferative potential *in vitro*[26-31]. There is thus strong evidence that stem cells in glandular tissues seem to have common characteristics and are hallmarked by their prominent nestin expression. All of these scientific results regarding the mammary gland are of paramount importance, as they unravel the basic principles of glandular development and homeostasis [20,21].

In view of an analogous embryonic gland development for mammary glands and sweat glands from ectodermal invaginations, it is of specific importance if there are also comparable characteristics between the isolated cell populations *in vitro*[32]. Hence, we applied isolation procedures and propagation methods for the harvesting of mammary stem cells similar to those we have already established for pancreatic, submandibular or sweat gland-derived stem cells [26,29,33]. We opted to isolate stem cells from the mammary tissue of female and male donors via adherence to conventional cell culture plastic, similar to the methods we used for the other glandular stem cells and also similarly applied by Cregan and colleagues for breast milk-derived stem cells [21]. To our knowledge we demonstrate here for the first time a method for the isolation and propagation of nestin-positive mammary gland-derived stem cells and present an initial characterization of the cell populations derived from both genders. This may foster the insights regarding mammary stem cells and can offer a relatively easy accessible source for (autologous) adult stem cell applications.

## Materials and methods

### Breast tissue biopsy collection

We received postoperative samples in conventional Ringer solution on ice and harvested them within 12 hours after tissue removal from the patient. All samples derived from breast or abdominal reduction surgeries (male patients) without pathological phenotype. Excluding the isolation establishment period, we were able to generate stable cell populations meanwhile from five female and three male donors up to passage 22 with the final standard protocol. The experiments of this study were performed with mammary gland-derived cells (MGDCs) from four different donors: MGDC 1, female patient, 47 years old; MGDC 2, male patient, 18 years old; MGDC 3, female patient, 40 years old; and MGDC 4, female patient, 50 years old. All experiments were performed according to Helsinki guidelines, in compliance with national regulations for the experimental use of human material. Utilization of human biopsies for research purposes was approved by the ethics committee of the University of Luebeck (reference number 10–058). All patients gave written informed consent.

### Isolation of acinary structures from human mammary tissues

During isolation we excised the small, pearl-like structures (diameter <2 mm) embedded in the mesenchymal parts of human breast tissue using forceps and scalpels. We manually freed them from adjacent fat and subsequently dissected and digested them using the patented method as described previously by Kruse and colleagues [27,28]. Briefly, after an initial mechanical dissection with scissors, the tissue was treated twice (20 and 15 minutes) with digestion medium containing HEPES–Eagle medium (pH 7.4), 10 mM HEPES buffer, 70% (v/v) modified Eagle’s medium, 0.5% (v/v) Trasylol (Bayer AG, Leverkusen, Germany), 1% (w/v) bovine serum albumin (PAA, Cölbe, Germany), 2.4 mM CaCl_2_ and collagenase (0.63 PZ/mg; Serva, Heidelberg, Germany) at 37°C. Prior to each digestion, the preparation mixture was gas-flushed with a mixture of oxygen and carbon dioxide (95% v/v). Between the two digestion steps, the tissue pieces were washed with isolation medium, being equal to digestion medium but lacking collagenase. Further processing was done by mechanical mincing using small chirurgic scissors. After the second digestion step, the remaining tissue fragments were dissociated by up-and-down suction through different glass pipettes with progressively more restrictive orifices (10, 5 and 2 ml pipettes) and filtered through a nylon mesh (250 μm) to exclude tissue debris. The acini-containing suspension was finally centrifuged for 5 minutes at 150 × *g* and the pellet was resuspended in DMEM (Invitrogen, Darmstadt, Germany) with 20% (v/v) FCS (PAA, Cölbe, Germany) and penicillin/streptomycin (PAA, Cölbe, Germany). The acini-containing medium was seeded into one well of a six-well cell culture test plate (TPP, Trasadingen, Switzerland) and this primary culture was incubated for 2 days in a humidified incubator with 37°C and 5% CO_2_ in the atmosphere. After 2 days the first media exchange was performed and the cultivated cells were propagated until highly confluent colonies were visible.

### Cultivation of mammary cells

The cultivation after the first trypsinization was performed with DMEM with 10% (v/v) FCS and penicillin/streptomycin in TPP cell culture plastic. We generally used polystyrene-plastic dishes as delivered (all cell culture treated by the supplier TPP). The incubator settings were the same as already described. When the population reached a confluence of >80% covered growth area, it was split in a ratio of 1:3 regarding the growth area by standard trypsinization. This includes the removal of the old medium, one washing step with PBS (Invitrogen, Darmstadt, Germany) and incubation for 2 minutes at 37°C after adding the Trypsin–ethylenediamine tetraacetic acid mixture (PAA, Cölbe, Germany). By microscopic control of the cell’s detachment, the stopping procedure starts by adding the doubled volume media to the trypsin-based suspension. The whole liquid was afterwards centrifuged at 180 × *g* for 5 minutes and the pellet was resuspended with cultivation medium. The cells were then reseeded to new cell culture plastics.

### Cell counting and growth curve

For determination of cell numbers, we trypsinized the cells and centrifuged them as described above. The resuspended pellet was then diluted with lysis buffer and stabilization buffer according to the manufacturer’s protocol (both ChemoMetec, Allerød, Denmark) and the cell suspension was measured with a Nucleocounter (ChemoMetec, Allerød, Denmark). To perform growth curves we seeded 50,000 cells per well in DMEM (Invitrogen, Darmstadt, Germany) with 10% (v/v) FCS (PAA, Cölbe, Germany) and penicillin/streptomycin in six six-well cell culture plates, using three wells of each plate (*n* = 3). At days 1, 3 and 5 the medium of one six-well plate was removed and cells were trypsinized as described before. Afterwards, 50 μl samples were measured using the Nucleocounter (ChemoMetec, Allerød, Denmark) and the arithmetic average was calculated.

### Time-lapse microscopy

The initially isolated acini were examined using time-lapse microscopy according to settings described in Rapoport and colleagues [30]. Briefly, we seeded an aliquot of the acini-containing suspension in a well of a six-well plate and cultivated the plate inside the culture chamber combined with an Olympus ix81 microscope (Olympus, Hamburg, Germany). Every 7.5 minutes a phase-contrast picture of the acini was taken automatically using 100× total magnification.

### Flow cytometry

For flow cytometry the cells were trypsinized, counted and pelleted according to the previously described cultivation protocol. Subsequently, they were resuspended in ice-cold fluorescent-activated cell sorting buffer containing 1% human normal immunoglobulin (Privigen; CSL Behring, Hattersheim am Main) diluted in PBS. The cell suspension was apportioned in 100,000 to 150,000 cells per well in a 96-well plate for all desired antibodies, isotype and negative controls. The whole subsequent procedure was performed on ice or in a cooled centrifuge. The plates were then centrifuged in an Allegra®X-15R centrifuge (Beckmann Coulter, Krefeld, Germany) for 5 minutes at 200 × *g* for accumulation of the cells on the well bottom. Subsequently the blocking was performed with pure immunoglobulin for 1 to 2 minutes and the primary-labeled antibodies (Table 1) were added to the blocking solution in adequate volumes to reach a final concentration of 10 μg/ml for each antibody. After incubation for 1 hour on ice, the cells were washed twice with fluorescent-activated cell sorting buffer alternating with a centrifugation step and resuspension in buffer. An immediate measurement of the fluorescence intensities was then performed with a FACS Calibur flow cytometer (Becton Dickinson, Heidelberg, Germany) and the according CellQuestPro Software (Becton Dickinson, Heidelberg, Germany). For standardization the isotype controls were gated with a cross gate and the tests were analyzed using this gate and calculating the positive events by subtraction of the quadrant statistics.

**Table 1:** Antibodies used for flow cytometric analysis

**Antigen**	**Host and clonality**	**Label**	**Catalogue number**
CD9	Mouse IgG1κ	Phycoerythrin	555372
CD15	Mouse IgG1κ	PerCP-Cy5.5	560828
CD29	Mouse IgG1κ	Phycoerythrin-Cy5	559882
CD31	Mouse IgG1κ	Phycoerythrin	555446
CD44	Mouse IgG1κ	Phycoerythrin	555479
CD81	Mouse IgG1κ	Phycoerythrin	555676
CD105	Mouse IgG1κ	PerCP-Cy5.5	560819
None	Mouse IgG1κ	Phycoerythrin	555749
None	Mouse IgG1κ	Phycoerythrin-Cy5	555750
None	Mouse IgG1κ	PerCP-Cy5.5	550795

All antibodies from Becton Dickinson (Becton Dickinson, Heidelberg, Germany). PerCP, peridinin chlorophyll protein.

### Polymerase chain reaction

MGDCs of the appropriate passage were cultured in a petri dish and when they reached a confluence of approximately 80% they were washed once with PBS. After removal of PBS they were scraped with a cell scraper (TPP, Trasadingen, Switzerland) in 2 ml fresh PBS, pelleted by centrifugation for 5 minutes at 180 × *g* and the pellet was stored at −80°C until analysis. The isolation of the total RNA was performed using an RNeasy Plus mini Kit and the automated sample preparation system QIAcube (both QIAGEN, Hilden, Germany) according to the manufacturer’s protocols. The concentration of eluted RNA was measured using a NanoDrop Spectrophotometer ND-1000 (Peqlab, Erlangen, Germany). Subsequently 1 μg total RNA was used for reverse transcription with a QuantiTect reverse transcription kit (QIAGEN, Hilden, Germany) including a genomic DNA digestion step.

The following polymerase chain reaction was performed as duplicates with 1 μl cDNA for each primer in a 25 μl reaction volume of QuantiFast SYBR Green PCR kit (QIAGEN, Hilden, Germany) using a RealPlex^2^ thermocycler (Eppendorf, Hamburg, Germany). The primers (QuantiTect Primer Assays) are all commercially available and validated by QIAGEN (Table 2). Some primers are intron-spanning for exclusion of genomic transcripts in the electrophoresis. For visualization and quality control of the generated amplicons we used the capillary gel electrophoresis system QIAxcel (QIAGEN, Hilden, Germany). A quantitative analysis of the expression intensity of selected relevant transcripts was evaluated with regard to the housekeeping gene β-actin. The cycle threshold mean of duplicate samples for the analyzed transcript was calculated and set in relation to the cycle threshold mean of the housekeeping gene (cycle threshold mean of β-actin/cycle threshold mean of specific transcript).

**Table 2:** List of used primers

**Amplified transcript**	**QuantiTect Primer Assay**
β-Actin	Hs_ACTB_1_SG QuantiTect Primer Assay (200)
c-Myc	Hs_MYC_1_SG QuantiTect Primer Assay (200)
Oct4	Hs_POU5F1_va.2_SG SG QuantiTect Primer Assay (200)
Klf4	Hs_KLF4_1_SG QuantiTect Primer Assay (200)
Sox2 (sex determining region Y –box 2)	Hs_SOX2_1_SG QuantiTect Primer Assay (200)
Nanog	Hs_NANOG_2_SG QuantiTect Primer Assay (200)
CD 9	Hs_CD9_1_SG QuantiTect Primer Assay (200)
Nestin	Hs_NES_2_SG QuantiTect Primer Assay (200)
Ki-67	Hs_MKI67_1_SG QuantiTect Primer Assay (200)
MEF2D (myocyte enhancer factor 2D)	Hs_MEF2D_2_SG QuantiTect Primer Assay (200)
α SMA (alpha-smooth muscle actin)	Hs_ACTA2_1_SG QuantiTect Primer Assay (200)
PPARγ (peroxisome proliferator-activated receptor gamma)	Hs_PPARG_1_SG QuantiTect Primer Assay (200)
SPP1 (secreted phosphoprotein 1)	Hs_SPP1_1_SG QuantiTect Primer Assay (200)
VWF (von Willebrand factor)	Hs_VWF_1_SG QuantiTect Primer Assay (200)
CK18 (cytokeratin 18)	Hs_KRT18_1_SG QuantiTect Primer Assay (200)
NF_l (neurofilament light chain)	Hs_NEFL_1_SG QuantiTect Primer Assay (200)
NF_m (neurofilament medium chain)	Hs_NEFM_1_SG QuantiTect Primer Assay (200)
NF_h (neurofilament heavy chain)	Hs_NEFH_1_SG QuantiTect Primer Assay (200)
Enolase (neuron-specific enolase)	Hs_ENO2_1_SG QuantiTect Primer Assay (200)
PGP 9.5 (protein gene product 9.5)	Hs_UCHL1_1_SG QuantiTect Primer Assay (200)
β3-Tubulin	Hs_TUBB3_1_SG QuantiTect Primer Assay (200)
MAP2 (microtubule-associated protein 2)	Hs_MAP2_1_SG QuantiTect Primer Assay (200)

All primers from QIAGEN (Hilden, Germany).

### Immunocytochemistry

The cells were washed once with PBS and fixed with methanol:acetone (7:3, v/v; both Carl Roth, Karlsruhe, Germany) and 1 mg/ml 4′, 6-diamidino-2-phenylindole (Roche, Mannheim, Germany) for 5 minutes at room temperature. After three washing steps with PBS they were blocked using 16.5 μl/ml normal goat serum (Vector Laboratories Ltd, Peterborough, UK) in PBS for 20 minutes at room temperature. The blocking mixture was then removed and the primary antibodies diluted in Tris-buffered saline Tween-20 buffer with 1% (w/v) BSA were added and incubated in a humidified chamber overnight at 4°C in a moist chamber. Primary antibodies raised against human nestin (mouse, monoclonal; Abcam, Cambridge, UK), Ki67 (rabbit, polyclonal; Abcam, Cambridge, UK), cytokeratin 19 (mouse, monoclonal; Sigma Aldrich, Taufkirchen, Germany), neurofilament light, medium and heavy chains (1:1:1 mixture, rabbit, polyclonal; AbD Serotec, Düsseldorf, Germany) and alpha-smooth muscle actin (mouse, monoclonal; Dako, Hamburg, Germany) were applied. After incubation, three washing steps were performed and the secondary antibodies were incubated under the same conditions as the primary ones. Secondary antibodies were Cy3-labeled goat-anti-mouse and fluorescein-labeled goat-anti-rabbit (both Dianova, Hamburg, Germany). After incubation for 1 hour at 37°C in a wet chamber, the secondary antibodies were removed by three washing steps with PBS and one rehydration step with distilled water. Samples were afterwards mounted with Roti-Mount Fluorocare mounting medium (Carl Roth, Karrlsruhe, Germany).

### Immunhistochemistry

Breast tissue was cut into a small piece of 0.5 cm edge length directly after delivery. After a brief rinse in fresh ice-cold PBS, the tissue was fixed for 1 hour in 4% (w/v) paraformaldehyde (Merck, Darmstadt, Germany) in PBS at 4°C. Subsequently the tissue was embedded in a handmade aluminum foil cube filled with OCT Tissue-Tek (Sakura, Alphen aan den Rijn, The Netherlands) and directly frozen at −80°C. For slicing we used a Cryotome (HM 560; Thermo Fisher Scientific, Schwerte, Germany) and prepared 5 μm thick slices, which were mounted on Menzel Superfrost Plus Gold slides (Menzel, Braunschweig, Germany). The subsequent storage until staining was at −20°C.

For staining we used a similar protocol to that described above. Initially the mounting media was rinsed away in distilled water for 2 minutes. Then the slice was fixed again with 4% paraformaldehyde solution for 5 minutes at room temperature. After three washing steps with PBS (each 5 minutes), the slices were encircled with a PAP hydrophobic barrier pen (Abcam, Cambridge, UK) and the blocking mixture was added to the circle’s center after drying of the line. The blocking procedure was performed for 20 minutes at room temperature, followed by incubation with the first antibody against human nestin (mouse, monoclonal; (Abcam, Cambridge, UK) overnight at 4°C in a moist chamber. After rinsing three times with PBS the secondary antibody, Cy3-labeled goat-anti-mouse (Dianova, Hamburg, Germany), was added and incubated for another hour under the same conditions. The subsequent three washing steps with PBS include in the first step incubation with 1 mg/ml 4′, 6-diamidino-2-phenylindole (Roche, Mannheim, Germany) for 5 minutes at room temperature. Finally, we rinsed the specimens in distilled water and mounted them using Roti-Mount Fluorocare mounting medium (Carl Roth, Karlsruhe, Germany).

## Results and discussion

### Mammary-residing nestin-positive cells can be isolated from tissue of both sexes

**Figure 1:**
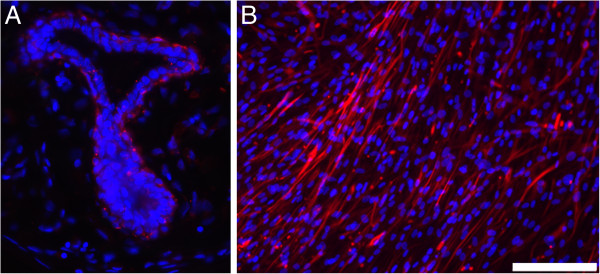
**Nestin expression in mammary tissue and derived cell populations.** Nestin-positive cells **(A)** in the female mammary gland and **(B)** in cultivated cells of mammary gland-derived cells (MGDC 3) after nine passages. Nestin was stained using a mouse monoclonal anti-nestin antibody (red). Nuclear counterstaining with 4′ , 6-diamidino-2-phenylindole (blue). Magnification: 200×. Bar = 100 μm.

The immunohistological examination of human frozen mammary tissue sections using a human specific anti-nestin-antibody revealed nestin-positive cells within the glandular structures. The majority of positive cells were observed in the peripheral layer of myoepithelial cells (Figure 1A). The localization of nestin-positive cells in the myoepithelial layer of mammary glands was previously described by Li and colleagues and Kolar and colleagues [23,24].

We were able to isolate cells from this tissue and propagate them over several passages (so far >20 passages) with maintenance of their nestin expression (Figure 1B). Cregan and colleagues have already published the isolation of nestin-positive cells from human breast milk [21]. Our observations revealed that the expression of nestin was not ubiquitous but in the majority of cells comparable with Cregan and colleagues’ results. We were additionally able to demonstrate the maintenance of this protein expression over several passages. Interestingly, comparable findings from our group concerning stem cells derived from the pancreas, salivary gland and sweat gland [26,27,29,33] revealed nestin expression as a common feature of gland-derived stem cells.

**Figure 2:**
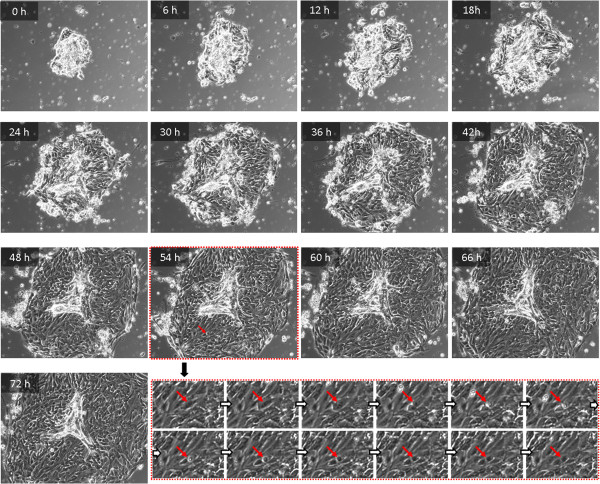
**Time-lapse microscopy of isolated mammary acinus.** Time-lapse micrographs (interval: 6 hours) of primary mammary cells arising from a single acinus. The lower panel (red dashed margin) illustrates the mitosis (interval: 7.5 minutes) at 54 hours indicated by the red arrow in each picture. Magnification: 100 ×.

**Figure 3:**
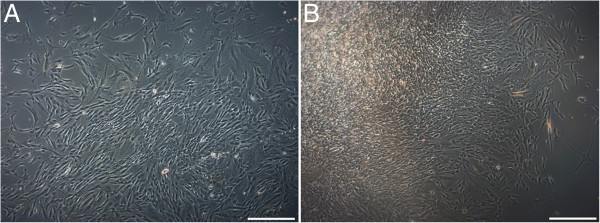
**Morphology of cultivated female and male mammary cell populations.** Primary population of **(A)** female mammary gland-derived cells (MGDC 3) and **(B)** male mammary gland-derived cells (MGDC 2) before first passaging. Magnification: 50×. Bar = 100 μm.

In view of these results we have to ask from which part of the tissue these cells originate. The continuous monitoring of the cell’s behavior during the outgrowth with time-lapse microscopy should provide insights into the acinar disintegration process, and consequently into the growth behavior of outgrowing glandular cells. We observed a continuous migration of cells out of the acinar structure and their stringent connection to each other as soon as they form a proliferating monolayer on the cell culture plastic. Proliferation activity and active movement was exclusively visible inside a sharp margin formed by elongated cells in the periphery of the colonies (Figure 2). After several days these colonies break up and the cells spread over to the remaining growth area, continuing their proliferation (Figure 3 and Additional file 1). For human pancreatic tissue a similar disintegration process of acinary structures and the establishment of a proliferating stem cell population were demonstrated by Rapoport and colleagues [30]. They characterized the apparent melting process of the acini with a fast migration of the cells on the cell culture surface ongoing with a rapid creation of a cell monolayer. Interestingly, no mitotic activity could be detected in their initial cell population.

We observed for the acinary structures from mammary glands a comparable process that shows one remarkable difference: the outgrowing cells proliferate in a tight colony bounded by elongated cells. This cell association breaks up after approximately 3 days and a slow emigration of singular but still proliferating cells was visible. So far we have no complete explanation for this specialty. We believe that this early process requires a tight intercellular connection and communication between the cells. The margin is probably formed by the first outgrowing cells and cannot be maintained for longer than 3 days. Ultimately, we generated a proliferating cell population, which is also described for the other glandular stem cell populations in later passages [30]. We therefore focused on the more detailed characterization to achieve closer insights into the stem cell characteristics in comparison with the previously described glandular stem cell populations. This proof-of-concept trial will reveal the possibilties to easily isolate adherently proliferating mammary cell populations with stem cell characteristics.

### Nestin-positive cells of both sexes proliferate adherently *in vitro*

**Figure 4:**
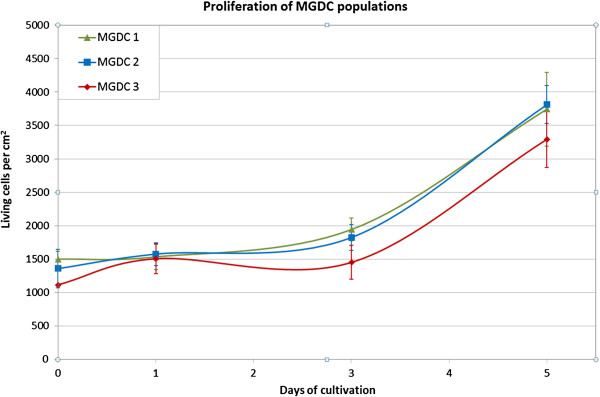
**Growth of mammary gland-derived cell populations *in vitro*.** Growth curves of two female mammary-gland-derived cell populations (MGDC 1, MGDC 3) and one male (MGDC 2) cell population in passage 7 over a cultivation period of 5 days. The mean cell amount of triplets was calculated for each cell population and time point (*n* = 3).

We succeeded in obtaining stably proliferating mammary cell populations from male and female tissue donors. The cells had a spindle-like shape and there were no obvious differences with regard to morphology or growth behavior between the cell populations of both genders (Figure 3). The standardized propagation of the mammary cells was so far possible until passage 22. For the three MGDC populations we examined growth behavior by cell counting and calculated growth curves over a time period of 5 days (Figure 4). After seeding, the cells needed an adaption phase to adjust to the new environment and started with a phase of exponential growth approximately at day 3. The population size grows continuously and we estimated a population doubling time during the exponential growth phase of approximately 2 days. We never observed multilayered growth or floating colonies during the daily monitoring of the cultivation routine. This observation indicates a healthy, contact-inhibited and anchorage-dependent population.

### Nestin-positive cells of both sexes have a phenotype comparable with other glandular stem cells

To classify the novel mammary cell population regarding their cell surface protein expression we additionally performed a flow cytometry analysis for one male sample (MGDC 2) and two female samples (MGDC 1 and MGDC 3) in passages 6 and 12. We selected different antibodies classically used in phenotypization of mesenchymal stem cells, which were also applied for characterization of gland-derived stem cells from the pancreas and salivary gland [26]. Similarly to Gorjup and colleagues, we obtained positive results for CD9 (tetraspanin-29), CD29 (integrin β1), CD44 (hyaluronan receptor), CD81 (TAPA-1) and CD105 (endoglin) [26] (Additional file 2). We observed for all populations and all passages qualitatively the same marker expression. Quantitatively, varying amounts of positively stained cells between the two passages 6 and 12 showed the same tendencies for all individuals.

**Figure 5:**
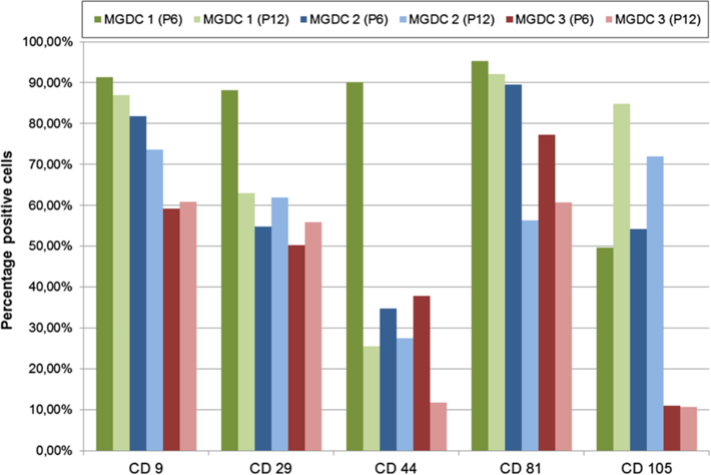
**Phenotypization of mammary gland-derived cell populations.** Analyses were performed with mammary gland-derived cell populations from three donors (MGDC 1, MGDC 2, MGDC 3) in passages (P) 6 and 12. The quantification of positive events for CD9, CD29, CD44, CD81 and CD105 were calculated for each cell population to show possible variations between genders and individuals.

Comparing the different individuals in each passage, a qualitative difference for the protein detection was obvious. As the donors’ sexes and ages differ, we assume that this is due to variable quantities of different phenotypic subpopulations (Figure 5). Nevertheless, we were able to show the common general expression of the positive surface antigens for each population of male and female individuals. The antigens CD15 (fucusyltransferase 4) and CD31 (PECAM-1) were not detectable or were <1% and thus were considered absent. The constitutive high yield of cells positive for CD9, CD29 and CD81 indicates the existence of stem cells in our populations, as also observed by Shackleton and colleagues and Gorjup and colleagues [3,26]. The occurrence of CD44^+^ cells in all populations points to the existence of possible migratory, duct-forming cells [34]. We observed a reduction in the amount of CD44^+^ cells over several passages. Whether this is due to apoptosis or dedifferentiation, transdifferentiation or normal differentiation is unratable at the moment and could therefore also be associated with a change in marker expression during *in vitro* culture. Notwithstanding, we assume that the cells do not become neoplastic because this would have been indicated through an upregulation of CD44 [34]. These observations support the findings of our proliferation analysis, regarding a stable and healthy population.

The expression of endoglin (CD105) is reported in a controversial context regarding mammary cells and tissues. This marker is usually included in tumor formation studies and indicates angiogenetic events because endoglin serves as a main surface protein for (endothelial) cell invasion by binding of integrins. Consequently, contrasting literature regarding endoglin synthesis by mammary cells exists describing protumorigenic and antitumorigenic involvement of this integrin-binding transforming growth factor beta receptor [35,36]. Regarding our recent results, we primarily reduce this information to the result that our mammary cell populations express CD105 in the context of *in vitro* cultivation for attachment to integrins. Liao and colleagues described endoglin as an essential marker for isolation of murine mammary stem cells that are able to form mammospheres. In the context of the basal function of CD105 for cells, their findings support our assumption because transplanted cells of their mammospheres resulted in a reconstitution of the mammary gland and not in tumor formation [37]. The lack of CD15 and CD31 indicates that there are no immune or endothelial cells present that may be a source for CD105^+^ cells. The determined cell surface phenotype of mammary cells *in vitro* seems to be comparable with other propagable stem cells from different glands and is similar to that of mesenchymal stem cells [26,38].

### Nestin-positive cells show expression of markers representative of the three germline layers

**Figure 6:**
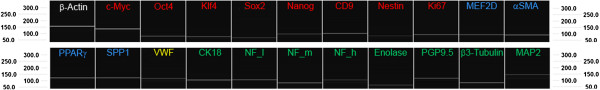
**Expression analysis of gene transcripts from mammary gland-derived cell populations.** Representative visualization of the capillary gel electrophoretic analyses of detected amplicons in the propagated female mammary gland-derived cells (MGDC 1) from passage 9. Expression of transcripts for the housekeeping gene β-actin (white), typical transcripts for stemness and proliferation (red) as well as for the mesodermal lineage (blue), the endodermal lineage (yellow) and the ectodermal lineage (green) could be verified.

**Figure 7:**
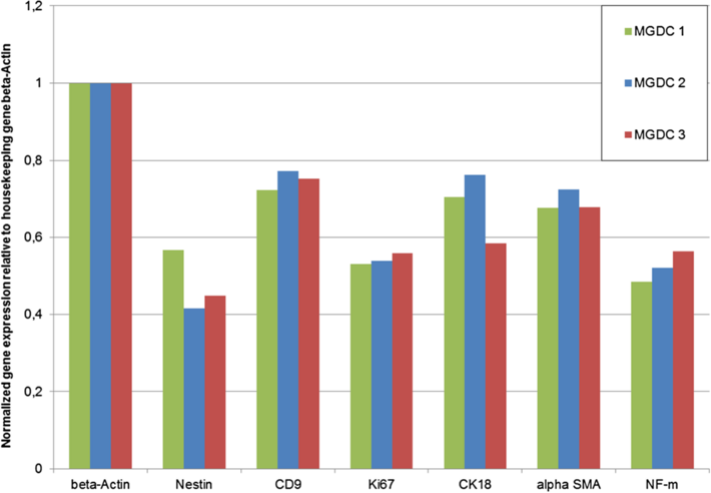
**Quantitative real-time PCR analyses of relevant transcripts in three mammary gland-derived cell populations.** Expression intensities of the analyzed transcripts for stemness (nestin, CD9), proliferation (Ki67) and differentiation (cytokeratin (CK) 18, alpha-smooth muscle actin (αSMA), neurofilament (NF)-m) were calculated from cycle threshold values (mean of duplicates) and set in relation to the housekeeping gene (cycle threshold mean of β-actin/cycle threshold mean of specific transcript).

**Figure 8:**
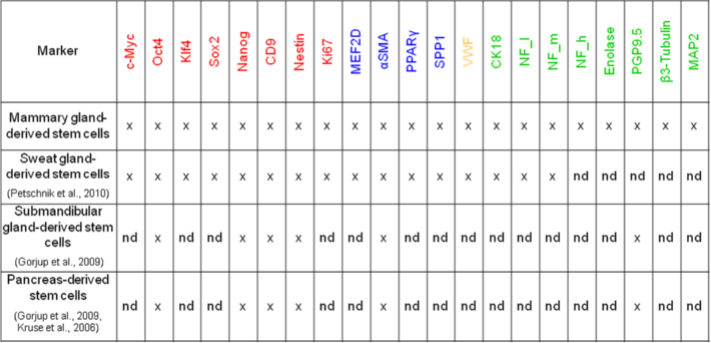
**Comparison of human gland-derived stem cells.** Table of the comparative gene expression analysis of mammary gland-derived cells (MGDCs) with glandular stem cell populations from the pancreas, salivary gland and sweat gland. Blue crosses, newly introduced primer for examination of the corresponding gene expression. nd, not determined.

For characterization of the cell populations with regard to a possible multipotent differentiation potential, we performed a gene and protein expression analysis from MGDC 1, MGDC 2 and MGDC 3 in passages 3 and 9 (*n* = 3). Using real-time PCR we examined transcripts indicating stemness (c-Myc, Oct4, Klf4, Sox2, Nanog, CD9, nestin) and proliferation (Ki67) as well as representative transcripts for estimating spontaneous differentiation. We successfully detected transcripts for all three embryonic germ layers (endodermal: von Willebrand factor; mesodermal: MEF2D, alpha-smooth muscle actin, PPARγ, SPP1; ectodermal: cytokeratin 18, neurofilament-light chain, neurofilament-medium chain, neurofilament-heavy chain, neuron-specific enolase, PGP9.5, β3-tubulin, MAP2) (Figure 6). To compare expression intensities between different male and female MGDC populations we quantitatively estimated the expression intensity of selected relevant transcripts (nestin, CD9, Ki67, cytokeratin 18, alpha-smooth muscle actin, neurofilament-middle chain) of MGDC 1, MGDC 2 and MGDC 3. Expression intensities fluctuate, but gross gene expression of the three analyzed cell populations MGDC 1 to MGDC 3 was similar (Figure 7). In comparison with the previous work of our group, this expression pattern is similar to those of human sweat gland-derived stem cells, submandibular gland-derived stem cells or the stem cell populations isolated from the pancreas (Figure 8) [26,29]. Even though we analyzed a wider panel of markers we can consider generally the mammary stem cells being similar to the other glandular stem cell populations regarding the common examined markers. The compared stem cell populations share the expression of genes for stemness, proliferation and all three germ layer derivatives.

**Figure 9:**
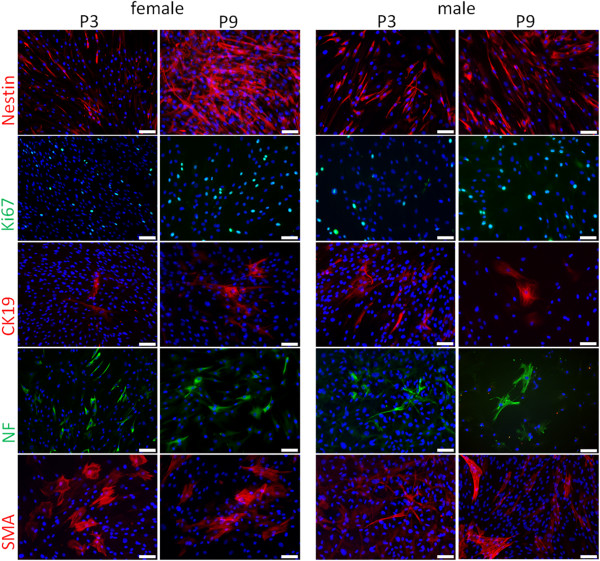
**Immuncytochemical analysis of expressed proteins in mammary gland-derived cells.** The staining for nestin (red), Ki67 (green), cytokeratin19 (CK19, red), neurofilaments (NF, green) and alpha-smooth muscle actin (αSMA) are exemplarily shown for a female mammary gland-derived cell population (MGDC 3, left panel) and a male mammary gland-derived cell population (MGDC2, right panel), each in passage 3 (left row of each panel) and passage 9 (right row of each panel). Nuclei are stained blue with 4′,6-diamidino-2-phenylindole. Bar = 100 μm. Magnification: 200 ×.

Within the scope of the results of the gene expression analysis, we checked our cell population for multilineage differentiation by immunocytochemical staining for several typical proteins. We therefore compared female and male cells, each in passages 3, 6 and 9, regarding their protein expression referring to the transcripts found in RT-PCR. All samples showed a distinct staining for nestin (stemness), Ki67 (proliferation), cytokeratin 19 (stemness), neurofilament (neuronal differentiation) as well as alpha-smooth muscle actin (mesodermal differentiation). Other examined proteins (CK8, CK18, CK14, Sox2, Oct4, Nanog, VWF, PPARγ, GATA4) showed a fluctuating expression level and should be analyzed in more detail in further experiments (data not shown). The proteins stained with continuous presence in passages 3, 6 and 9 (representative results for passages 3 and 9 in Figure 9) showed less differences between the individuals or sexes.

To the best of our knowledge we are the first to show a consistent nestin expression for female and male cells derived from mammary glands over several passages. Our identification of cells positive for this adult stem cell marker was supported by the successful detection of cytokeratin 19, a considered marker for mammary stem cells, and the positive evaluation of various Ki67-synthesizing cells indicating proliferative activity [5,16,22]. Furthermore we demonstrated that the expression of the mesodermal protein alpha-smooth muscle actin and of the ectodermal neurofilaments is maintained in culture in male and female cell populations. We presume this is a result of a continuous, spontaneous differentiation of an unrestricted amount of stem cells.

We would like to emphasize that additional information about the nestin-positive cell population may foster the comprehension of MGDC populations with regard to their stemness. Furthermore, their possible multipotency, functionality and application shall be examined in *in vivo* studies to define the impact on possible therapeutic strategies. For reaching these goals, definitively more human samples are needed.

## Conclusion

With the current work we present an easy and reliable method to isolate nestin-positive stem cells from male and female mammary tissue. We monitored the formation of an adherent cell population from acinary mammary structures *in vitro* and succeeded in establishing stably proliferating mammary stem cell populations. During *in vitro* cultivation the mammary cell populations exhibit a spontaneous differentiation potential and give rise to cell types of endodermal, mesodermal and ectodermal origin. By adapting methods and techniques for the isolation of other gland-derived stem cell populations we gained comparable nestin-positive, multipotent stem cell populations from mammary gland tissue derived from both genders. These results may have impact for novel clinical strategies for cell-based therapies.

## Abbreviations

BSA: Bovine serum albumin; DMEM: Dulbecco’s modified Eagle’s medium; FCS: Fetal calf serum; K: Keratin; Klf4: Krueppel-like factor 4; MAP2: Microtubule-associated protein 2; MEF2D: Myocyte-specific enhancer factor 2D; MGDC: Mammary gland-derived cell; Oct4: Octamer binding transcription factor 4; PBS: Phosphate-buffered saline; PCR: Polymerase chain reaction; PGP 9.5: Protein gene product 9.5; PPARγ: Peroxisome proliferator-activated receptor gamma; RT: Reverse transcriptase; Sox2: Sex determining region Y – box 2; SPP1: Secreted phosphoprotein 1; VWF: Von Willebrand factor.

## Competing interests

The authors declare that they have no competing interests.

## Authors’ contributions

AR participated in the conception and design of the study, performed the experiments and drafted the manuscript. NN performed the experiments and analyzed the data. PM participated in the conception and interpretation of the study and contributed reagents, materials and analysis tools. FSt participated in data analysis and revised the manuscript critically. FSi contributed materials and reagents and participated in manuscript drafting. CK participated in the design of the study, analyzed the data and revised the manuscript. SD conceived the experiments, interpreted the data and gave final approval of the manuscript to be published. All authors read and approved the manuscript for publication.

## Supplementary Material

Additional file 1 **Time-lapse movie showing the *in vitro * cultivated mammary gland acinus.** The outgrowth of mammary gland cells from the acinar structure has been documented by time-lapse microscopy. Continuously observation was done by taking a picture every 7.5 minutes for a time period of 7 days. Mammary gland cells adhere to the cell culture plastic, migrate out of the acinar tissue and some single cells start to proliferate. Click here for file

Additional file 2 **Diagrams of fluorescent-activated cell sorting analysis for one exemplarily shown cell population (MGDC 1).** Representative graphs of flow cytometric analysis of a female mammary cell population (MGDC 1) in passage 6. The counted events per fluorescence intensity of the samples (purple area) are referred to that of the isotype control (green line) for each measurement. PE, phycoerythrin; PerCP, peridinin chlorophyll protein. Click here for file
