# Supplementary figures and images for: Mammary gland-derived nestin-positive cell populations can be isolated from human male and female donors
Source: Stem Cell Res Ther. 2013 Jul 8;4(4):78. doi: 10.1186/scrt229 (PMC3854770; doi:10.1186/scrt229)

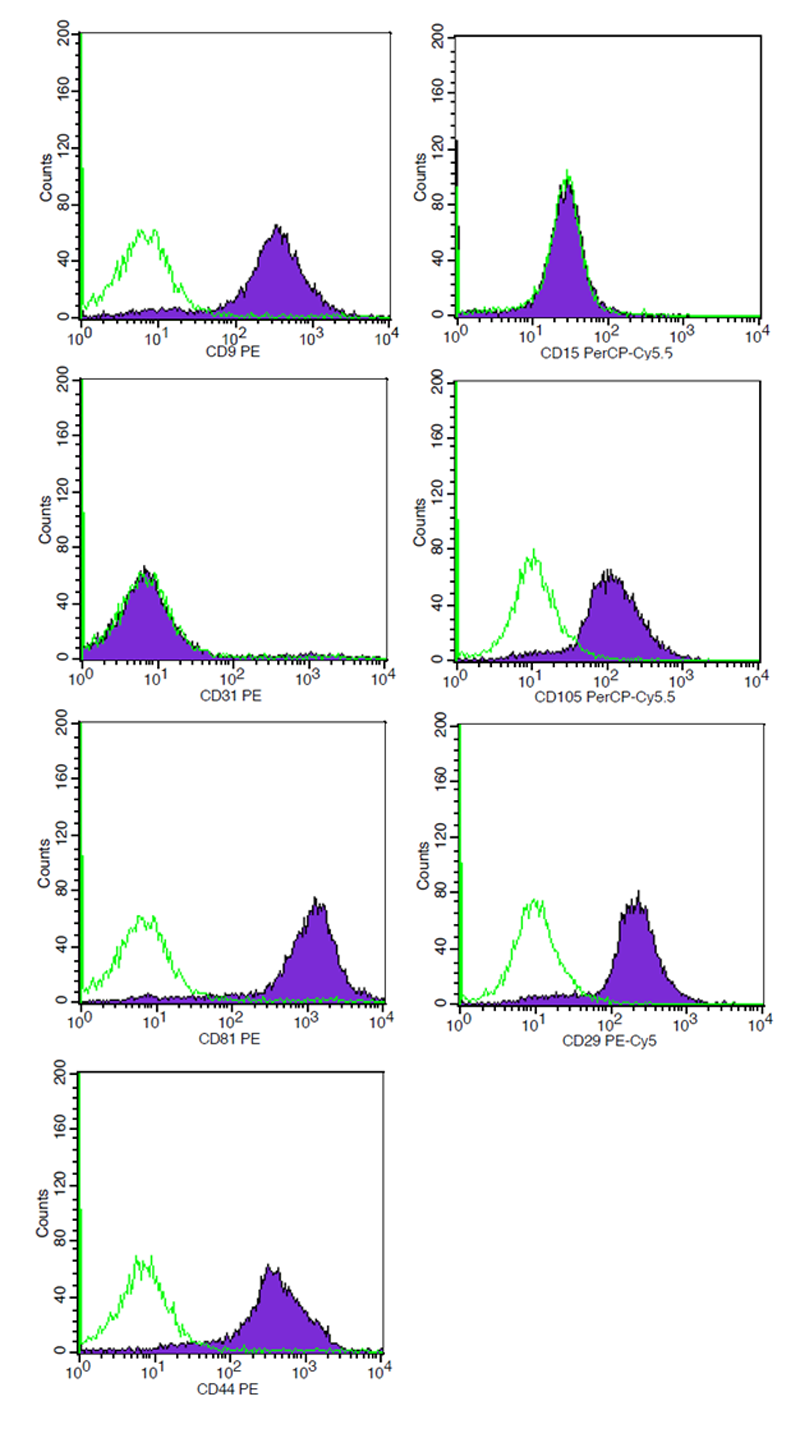

Supplement: Additional file 2 — Diagrams of fluorescent-activated cell sorting analysis for one exemplarily shown cell population (MGDC 1). Representative graphs of flow cytometric analysis of a female mammary cell population (MGDC 1) in passage 6. The counted events per fluorescence intensity of the samples (purple area) are referred to that of the isotype control (green line) for each measurement. PE, phycoerythrin; PerCP, peridinin chlorophyll protein. [file scrt229-S2.tiff]
